# The cellular and molecular determinants of emphysematous destruction in COPD

**DOI:** 10.1038/s41598-017-10126-2

**Published:** 2017-08-25

**Authors:** Masaru Suzuki, Marc A. Sze, Joshua D. Campbell, John F. Brothers, Marc E. Lenburg, John E. McDonough, W. Mark Elliott, Joel D. Cooper, Avrum Spira, James C. Hogg

**Affiliations:** 1 Centre for Heart Lung Innovation, St. Paul’s Hospital, Departments of Medicine, and Pathology and Laboratory Medicine, University of British Columbia, Vancouver, BC Canada; 2 Division of Computational Biomedicine, Department of Medicine, Boston University School of Medicine, Boston, MA USA; 3 Division of Thoracic Surgery, Hospital of the University of Pennsylvania, Philadelphia, PA USA; 4 Department of Respiratory Medicine, Faculty of Medicine and Graduate School of Medicine, Hokkaido University, Sapporo, Japan

## Abstract

The introduction of microCT has made it possible to show that the terminal bronchioles are narrowed and destroyed before the onset of emphysematous destruction in COPD. This report extends those observations to the cellular and molecular level in the centrilobular phenotype of emphysematous destruction in lungs donated by persons with very severe COPD (n = 4) treated by lung transplantation with unused donor lungs (n = 4) serving as controls. These lung specimens provided companion samples to those previously examined by microCT (n = 61) that we examined using quantitative histology (n = 61) and gene expression profiling (n = 48). The histological analysis showed that remodeling and destruction of the bronchiolar and alveolar tissue is associated with macrophage, CD4, CD8, and B cell infiltration with increased formation of tertiary lymphoid organs. Moreover, gene set enrichment analysis showed that genes known to be expressed by natural killer (NK), lymphoid tissue inducer (LTi), and innate lymphoid cell 1 (ILC1) cells, but not ILC2 or ILC3 cells, were enriched in the expression profiles associated with CD4, CD8, and B cell infiltration. Based on these findings, we postulate that the centrilobular phenotype of emphysematous destruction COPD is driven by a Th1 response activated by infiltrating ILC1, NK, and LTi cells.

## Introduction

The decline in lung function used to measure the progression of chronic obstructive pulmonary disease (COPD) has frequently been linked to the infiltration of lung tissues by polymorphonuclear leukocytes (PMNs), macrophages, CD4, CD8, and B cell lymphocytes^1–6^. In addition, studies from several laboratories have shown that this infiltration is associated with a sharp increase in tertiary lymphoid organ formation consistent with an adaptive immune response^5–7^. Although once considered a curiosity, this pattern of progressively increasing macrophage, CD4, CD8, and B cell lymphocyte infiltration with tertiary lymphoid organ formation is now recognized to be a fundamental pathological process that links the host inflammatory immune response to a destructive form of tissue repair^8^. This type of tissue response may be either beneficial to the host by combating invasive tumors and infections or detrimental to the host by initiating tissue destruction in many different organs^8^ and possibly peripheral lung tissue in COPD^5–7^. In addition, a recent review of the biology of the innate immune response by Artis and Spits^9^ suggests that the newly discovered innate immune lymphoid cells (ILCs) termed ILC1, ILC2, and ILC3, that have only recently been demonstrated in human lung tissue^10^, interact with innate immune lymphoid tissue inducer (LTi) cells and natural killer (NK) cells to deliver a potent innate immune stimulus to the adaptive immune response without the requirement for antigen presentation. Further, these new data also suggest that ILC1 cells preferentially stimulate a Th1 response, that ILC2 cells stimulate a Th2 response, and that the ILC3 cells stimulate Th17 and Th22 adaptive immune responses^9^. The purpose of this report is to examine the relationship between these infiltrating inflammatory immune cells and the remodeling and destruction of both bronchiolar and alveolar tissues in the earliest stages of emphysematous destruction before the lesions become large enough to be visualized on thoracic multi-detector computed tomography (MDCT) scans.

## Results

### Patient demographics

Table 1 shows the age, sex, smoking histories, and SaO_2_ for all 8 subjects as well as total tissue and gas volumes computed from MDCT specimen scans, the numbers of lung slices examined/specimen, numbers of samples examined/slice, and total number of tissue samples examined/lung specimen. In addition, the data also shows that the FEV_1_ and FEV_1_/FVC indicate all 4 of the subjects with COPD were in the very severe (GOLD 4) disease category.

**Table 1:** Subject demographics.

Patient ID	Control #1	Control #2	Control #3	Control #4	COPD #1	COPD #2	COPD #3	COPD #4
Phenotype	Donor	Donor	Donor	Donor	CLE	CLE	CLE	CLE
Number of cores	8	8	8	5	8	8	8	8
Sex	M	M	M	M	M	F	F	M
Age	51	59	62	43	62	63	56	59
Pack-years	39	0	24	0	50	38	54	30
FEV_1_ (% predicted)	N/A	N/A	N/A	N/A	21	12	24	15
FEV_1_/FVC (%)	N/A	N/A	N/A	N/A	22	26	24	35
SaO_2_ (%)	99	99.5	99	99.8	71	85.4	97.2	98
Lung volume (ml)	2826	3227	2959	3992	3053	2169	3559	5042
Gas volume (ml)	2522	2890	2670	3673	2671	1899	3234	4675
Tissue volume (ml)	304	337	289	318	382	270	325	368

CLE = centrilobular emphysema, FEV_1_ = forced expiratory volume in one second, FVC = forced vital capacity, SaO_2_ = arterial oxygen saturation, N/A = data not available.

### Quantitative histology

**Figure 1:**
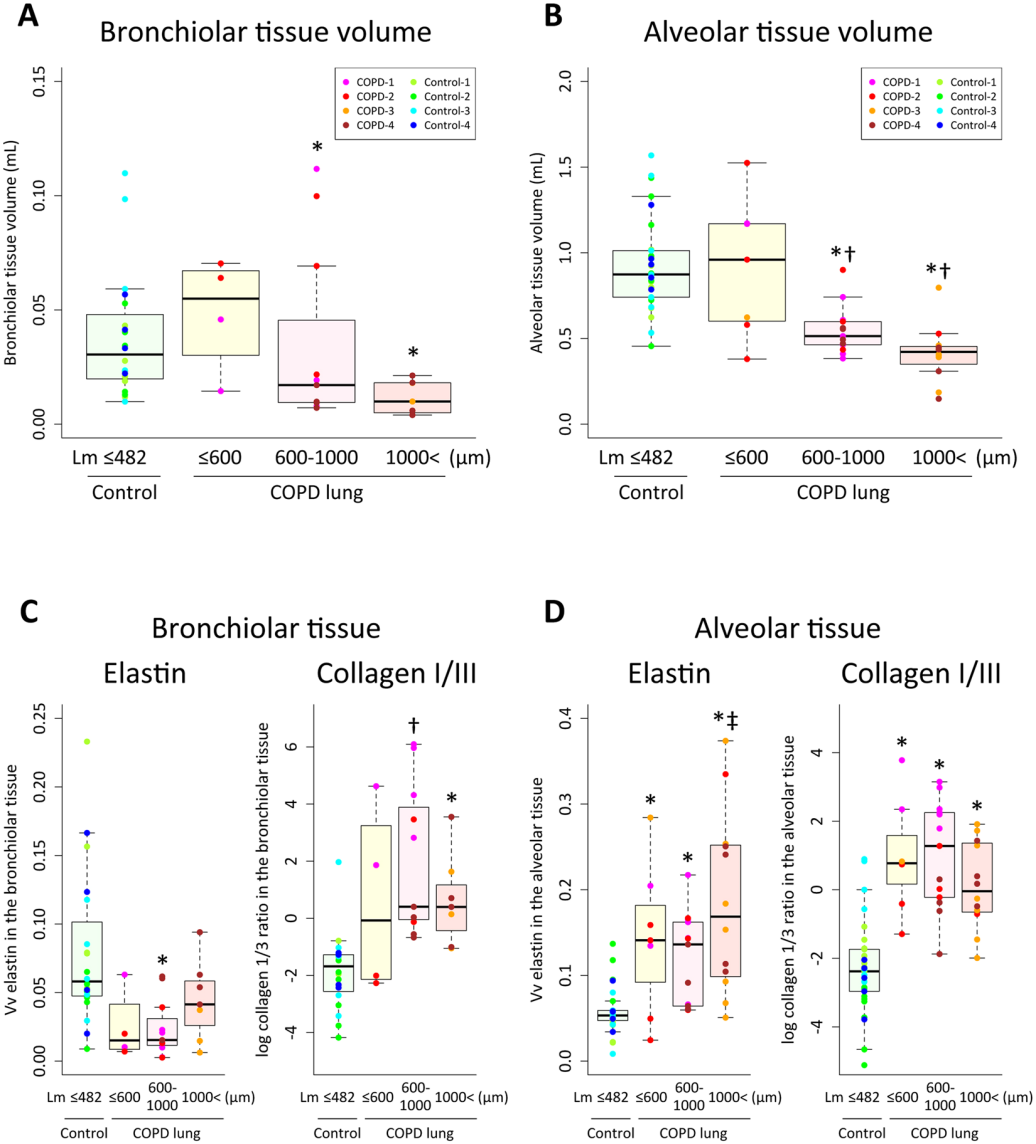
The changes in bronchiolar tissue volume of lung cores that contained small airways (n = 42) (**A**), alveolar tissue volume of all lung cores (n = 61) (**B**), and volume fractions of elastin and collagen I/III ratio in the bronchiolar tissue (**C**) and the alveolar tissue (**D**). Each color code represent tissue samples from each patient. *p < 0.05 versus control, ^†^p < 0.05 versus Lm ≤ 600 µm, ^‡^p < 0.05 versus 600 < Lm ≤ 1000 µm by a linear mixed-effects model considering the subject as a random effect.

We estimated tissue volume, volume fraction (Vv) of elastin, collagen I and III, and inflammatory cells both in bronchiolar and alveolar tissues using a multi-level sampling design. Figure 1A and B compare the changes in total bronchiolar and alveolar tissue volumes at different stages of emphysematous destruction. There was a small statistically insignificant increase in both bronchiolar and alveolar volumes in the diseased lungs when Lm remained below the upper limit of normal observed in control lungs (600 µm)^11^ compared to the control donor lungs (Lm ≤ 482 µm in this study). Then, both bronchiolar and alveolar tissue volumes decreased as emphysematous destruction progressed. As expected from earlier publications^12, 13^, Vv of elastin decreased in bronchiolar tissue and increased with abnormal appearance in alveolar tissue compared to control lungs (Fig. 1C,D, Supplementary Figure S1A) as Lm increased. Furthermore, this destructive remodeling process is associated with a significant increase in ratio of collagen I to III in both tissues compared to the control lungs (Fig. 1C,D, Supplementary Figure S1B,C). These findings are consistent with scar formation in these tissues before the emphysematous lesions became large enough to be visible on thoracic MDCT scans.

**Figure 2:**
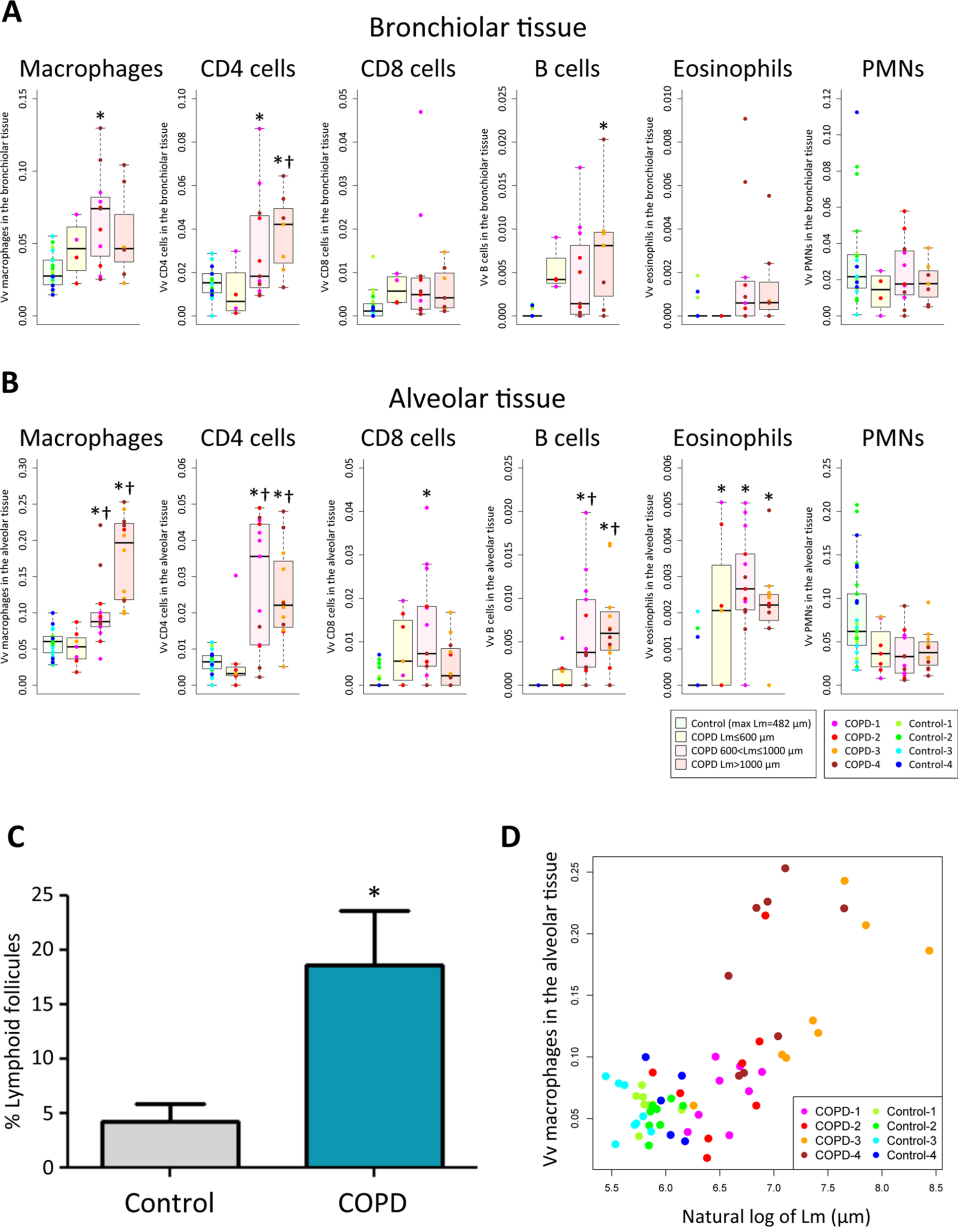
The changes in volume fractions of macrophages, CD4 cells, CD8 cells, B cells, eosinophils, and PMNs in the bronchiolar tissue (**A**) and the alveolar tissue (**B**). *p < 0.05 versus control, ^†^p < 0.05 versus Lm ≤ 600 µm by a linear mixed-effects model considering the subject as a random effect. (**C**) Average percentage of airways and vessels with lymphoid follicles. *p < 0.05 versus control. (**D**) Relationship between volume fraction (Vv) of macrophages in the alveolar tissue (alveolar wall + alveolar space) and the severity of emphysematous destruction (natural log of Lm). Each color code represents tissue samples from individual patient.

Figure 2A shows that when the inflammatory immune cell infiltration into bronchiolar tissue was compared to control lungs, Vvs of macrophages, CD4 cells, and B cells in bronchiolar tissue were increased in diseased lungs compared to control lungs. And Fig. 2B shows that Vvs of macrophages, CD4 cells, CD8 cells, B cells, and eosinophils in alveolar tissue were increased in emphysematous lung tissue compared to the controls. Collectively, these findings suggest that the destructive remodeling of both in bronchiolar and alveolar tissues is associated with progressive infiltration by macrophages, CD4, and B cells with additional infiltration of the alveolar tissues by CD8 cells and eosinophils. Supplementary Figure S2 provides representative images of these changes and a complete summary of all the primary data is provided in Supplementary Table S3. In addition, the analysis of the histology confirmed the increased formation of tertiary lymphoid organs in the diseased lungs compared to the control lungs (Fig. 2C), as well as a close association between the increase in Lm and Vv of infiltrating macrophages in alveolar tissue (Fig. 2D).

Next, we performed a random forest analysis with the Boruta selection feature^14^ to determine which of the microCT and histological measurements best predicted the increase in Lm. The results of the random forest analysis (Table 2) confirmed the initial analysis by showing that the infiltration of the tissues by CD4 cells, B cells, macrophages, and eosinophils, as well as the reduction in numbers of terminal bronchioles, and increase in collagen I were the best predictors of the increase in Lm observed in this study. Further, when all of these components were reanalyzed using a multivariate linear mixed-effects model, the infiltration of macrophages (p = 0.01), CD4 cells (p = 0.05), and B cells (p = 0.006) into the alveolar tissue were associated with the increase in Lm (see Supplementary Table S4).

**Table 2:** The variables that predict an increase in Lm based on a random forest analysis using the Boruta feature selection.

Variable tested	Ranked importance	Direction	Importance measure
Vv B cells (alveolar tissue)	1	Positive	10.26
Vv B cells (alveolar & bronchiolar average)	2	Positive	10.18
Vv macrophages (alveolar wall)	3	Positive	8.65
Vv CD4 cells (alveolar tissue)	4	Positive	8.59
Vv macrophages (alveolar & bronchiolar average)	5	Positive	7.66
Vv collagen I (alveolar wall)	6	Positive	6.76
Vv macrophages (alveolar tissue)	7	Positive	6.76
Number of terminal bronchioles/ml	8	Negative	6.62
Vv CD4 cells (alveolar & bronchiolar average)	9	Positive	5.72
Vv eosinophils (alveolar tissue)	10	Positive	4.04
Vv B cells (bronchiolar tissue)	11	Positive	3.95

Vv = volume fraction. Information of all variables that were put into the Boruta feature selection is shown in the online supplement. Directionality was determined by Spearman’s rank correlation coefficient.

### Gene expression profiling

We associated gene expression profiling of whole lung tissue by mRNA microarray with histological measurements. Table 3 shows that macrophage infiltration is associated with the expression of 353 genes and that PMN infiltration is associated with the expression of 2467 genes (FDR < 0.10). Moreover, the chi-square analysis showed that 39 genes associated with macrophage infiltration, 15 genes associated with CD4 cell infiltration, and 20 genes associated with B cell infiltration were enriched in the previously reported 127 gene signature for emphysematous destruction^15^. In sharp contrast, only 1/2467 gene expressed in association with neutrophil infiltration was enriched in the 127 gene expression signature for emphysema. In addition, this analysis also showed that 14 genes were expressed in association with the infiltration of the tissue by more than one of these three infiltrating cells (see Supplementary Table S5).

**Table 3:** Genes associated with infiltration macrophages, CD4 cells, and B cells are enriched in a previously reported 127 gene expression profile associated with emphysematous destruction (Lm).

Gene expression correlation	FDR < 0.10	Similar to 127 genes in the gene expression profile for emphysema	Significance
Lm	127	127	
Macrophages	353	39*	p < 0.001
CD4 cells	0	15*	p < 0.001
CD8 cells	0	0	p = 0.94
B cells	0	20*	p < 0.001
Eosinophils	0	0	p = 0.94
PMNs	2467	1	p = 0.94

Lm = mean linear intercept. *Although the total number of genes in the expression profiles associated with macrophage, CD4, and B cell infiltration was 74, the total number included in the previously reported 127 gene expression signature is reduced to 58 because 14 of these genes were expressed by more than one cell (see list in Supplementary Table S5).

We also performed a gene set enrichment analysis (GSEA)^16^ to determine if the gene expression signatures associated with infiltrating inflammatory cells were enriched by genes in the published gene sets associated with innate immune cells and dendritic cells. This GSEA analysis showed (Table 4) that previously published lists of genes expressed by NK cells, LTi cells, and ILC1 cells were enriched in the expression profiles associated with CD4, CD8, and B cell infiltration, whereas the genes expressed by ILC2 cells were not enriched in any of these profiles, and the genes expressed by ILC3 cells were only enriched in the profile associated with infiltrating CD8 cells. On the other hand, the published list of genes expressed by dendritic cells was enriched in the expression profiles associated with PMN, macrophage, CD4, CD8, and B cell infiltration.

**Table 4:** Genes expressed by innate immune cells are enriched in genes associated with inflammatory cell infiltration.

Immune cell gene profile	Terminal bronchioles	Lm	PMNs	Macrophages	CD4 cells	CD8 cells	B cells
NK cells	—	—	—	—	0.007	0.08	0.09
LTi cells	—	—	—	—	0.08	0.09	0.08
ILC1	—	—	—	—	0.015	0.09	0.05
ILC2	—	—	—	—	—	—	—
ILC3	—	—	—	—	—	0.09	—
DC	—	—	0.01	0.003	0.002	< 0.001	0.05

Only FDR values at 0.10 or less are listed in the table and (—) indicates values above FDR cutoff of 0.10. NK = natural killer, LTi = lymphoid tissue-inducer, ILC = innate lymphoid cell, DC = dendritic cells, Lm = mean linear intercept.

## Discussion

McDonough *et al*.^11^ combined the analysis of MDCT scans of intact air inflated lung specimens with microCT studies of samples of tissue removed from these lungs. They showed that destruction of the terminal bronchioles precedes the onset emphysematous destruction measured by microCT and that the destruction of the terminal bronchioles is well established when the emphysematous lesions become large enough to be visualized on thoracic MDCT scans. The present results substantially extend these findings by providing quantitative information about the changes in histology and gene expression profiling associated with this progressive destruction of bronchiolar and alveolar tissues. For example, the modest trend towards an increase in both bronchiolar and alveolar tissue volume in regions of the diseased lungs where Lm remained within the upper limits of the range normal can be is attributed to swelling of these damaged tissues. Furthermore, the progressive reduction in both bronchiolar and alveolar tissues that occurred in diseased lungs after Lm increased beyond the upper limits observed in control lungs is consistent with earlier reports showing that the elastin content of the bronchioles is decreased^12^ and that there is an increase in elastin in alveolar tissue undergoing destruction^13^. In addition, the progressive increase in the collagen I/III ratio as Lm continues to increase is consistent with scar tissue formation during this destructive remodeling process^17^. Further, the association between the destructive remodeling processes observed in both bronchiolar and alveolar tissue with the infiltration of macrophages, CD4 cells, and B cells, with additional infiltration of the alveolar tissue CD8 lymphocytes and eosinophils (Fig. 2A and B) combined with increased formation of tertiary lymphoid organs (Fig. 2C), is consistent with the presence of an adaptive immune response. Moreover, the observation that the association between infiltrating cells and the increase in Lm was strongest for the macrophages and weakest for PMNs (Fig. 2A,B) plus the observation that this relationship between the increase in Lm and macrophage infiltration was maintained when based on individual cores of tissue rather than the entire lung (Fig. 2D) singles out the alveolar macrophage as the most probable phagocyte associated with emphysematous destruction in COPD. The random forest analysis of the entire data set (Table 2) confirmed that infiltrating cells plus the reduction in numbers of terminal bronchioles provide the best predictors for the increase in Lm. This strongly supports the hypothesis that there is a strong association between the destruction of terminal bronchioles in the centrilobular emphysematous phenotype of COPD and the presence of an adaptive immune response that activates macrophages to destroy the bronchiolar and alveolar tissue.

Remarkably, this within lung comparison of the changes in histology observed in association with an increase in Lm within individual lungs is surprisingly similar to a previously reported between lung comparison of the histological changes observed when lungs from smokers with normal lung function were compared to lungs from persons at all 4 stages of the GOLD classification of COPD severity^5^. Both studies showed the measurements of disease progression are associated with infiltration of the tissues by macrophages, CD4 cells, CD8 cells, B cells, and tertiary lymphoid organ formation. The major strength of the between lung comparison reported in 2004 is that it allowed disease progression to be linked to routine measurements of lung function obtained over the full range of the 4 stages of COPD severity. In contrast, the major strength of the present within lung comparison is that it allows the changes in histology and gene expression profiling to be compared on the unique genetic background and similar environmental exposures received by individual persons.

The gene expression profiles associated with infiltrating cells visible on histological examination were obtained using a modification of the approach developed by Campbell *et al.* ^15^ to determine the 127 gene expression signature associated with increasing Lm, where instead of inserting a value for Lm into equation 2 of the linear mixed effect model^15^, we inserted the Vv of the infiltrating cell of interest (see Supplementary Methods). The gene expression signatures associated with these each of these infiltrating cells was then compared to the previously reported 127 gene signature for emphysematous destruction^15^ (Table 3). These results showed that 39/353 genes associated with macrophage infiltration were enriched in the previously reported 127 signature for emphysematous destruction^15^, whereas only 1/2457 genes associated with PMN infiltration were enriched in this same signature. Further, this gene expression signature for emphysematous destruction is also enriched by 15 genes associated with CD4 cell infiltration and 20 genes associated with B cell infiltration. Collectively, these data provide preliminary support for the concept that the gene expression profiles associated with macrophage, CD4 cell, and B cell infiltration accounted for 58/127 (46%) of the 127 genes the previously reported expression signature for emphysematous destruction observed in these same tissues^15^. Moreover, the observation that 14 of these 58 genes were associated with more than 1 of these phenotypes of infiltrating cell suggests complex milieu of cytokines and chemokines required to drive this destructive process requires extensive co-operation between several different cell types. It is also of interest 5 of these 14 genes (i.e., WFDC1, ACVRL1, STARD13, OSBPL3, and BCL11A) have already been implicated in several different aspects of the tissue repair process. For example, WFDC1 is associated with the tissue repair that occurs in response to infection^18^, and ACVRL1, STARD13, OSBPL3, and BCL11A are associated with angiogenesis, hematopoietic cell differentiation, and B cell function, respectively^19–22^.

Although others have linked PMN, macrophage, and lymphocyte infiltration to a decline in lung function^1–7^, Finkelstein *et al*.^23^ were the first to directly demonstrate that emphysematous destruction is more closely associated with lymphocyte and macrophage rather than PMN infiltration. The present results substantially extend those earlier observations by showing that genes associated with infiltrating macrophages, CD4 and B cell lymphocytes account for 58/127 (46%) of the 127 gene expression signature previously associated with emphysematous destruction, and that the infiltrating macrophages account for 39/58 (67%) of the genes associated with infiltrating cells that are enriched in the 127 gene expression signature for emphysematous destruction. These observations add to the mounting evidence infiltrating macrophages are the most important phagocyte associated with destruction of both bronchiolar and alveolar tissue in the centrilobular phenotype of emphysematous destruction in COPD. Importantly, the rapidly expanding literature on macrophage function suggests that CD68 used to identify macrophages in this study probably stains several different macrophage phenotypes created by their surrounding cytokine milieu^24–26^. Moreover, *in vivo* studies of the repair of full thickness skin wounds in genetically modified mice have shown that depleting the macrophages at different time points during this repair process results in remarkably different outcomes^27^. These findings strongly support the hypothesis that different phenotypes of alternatively activated macrophage may emerge to control different features of this repair process. On the other hand, CD68 has been reported to be not specific for macrophages^28–30^, and it was shown to be also expressed by fibroblasts in airway wall^31^. Although the quantification of macrophages was performed based on both of CD68-positivity and typical cell morphology in this study, incidental inclusion of the CD68-positive fibroblasts could be one of the limitations of this study.

Finally, Table 4 provides preliminary data relevant to the explosion of interest in the innate immune system recently reviewed by Artis and Spitz^9^ by using existing lists of the gene expression profiles associated with the relatively newly discovered innate immune lymphoid cells termed ILC1, ILC2, and ILC3 that have only recently been demonstrated in human lung tissue^10^, in addition to other lists of LTi cells, NK cells, and dentritic cells that are thought to deliver a potent innate immune stimulus to the adaptive immune response to antigens that were previously ignored by the host immune system. Moreover, Artis and Spits have reviewed the evidence that ILC1 cells preferentially stimulate a Th1 response, that ILC2 cells preferentially stimulate a Th2 response, and that the ILC3 cells preferentially stimulate Th17 and Th22 adaptive host immune response^9^. We used GSEA analysis (Table 4) to show that genes known to be expressed by NK, LTi, ILC1, and dendritic cells were enriched in the expression profiles associated with infiltrating CD4, CD8, and B cell lymphocytes, whereas genes known to be expressed by ILC2 cells were not enriched in the expression profiles associated with any of these cells, and the genes expressed by ILC3 cells were only enriched in the expression profile associated with infiltrating CD8 cells. These results provide preliminary evidence in support of the concept that ILC1, NK, LTi, and dendritic cells may be responsible for the activation of the Th1 response associated with emphysematous destruction that has been previously reported by several different laboratories^32, 33^. Moreover, we find the hypothesis particularly attractive because it has the potential to explain several very poorly understood features of the pathogenesis of COPD. For example, the well-established observation that only a “susceptible minority” of smokers develops the rapid decline in lung function that leads to the early onset COPD might be explained if innate immune stimulation could only induce this type of Th1 response to microbial antigens that emerged as the microbiome loses its diversity in COPD^34^. Further, auto antigens to such as abnormal elastin that might appear during either abnormal lung development or an abnormal tissue repair process^35, 36^ might induce a host response that prevented the lung from developing normal maximal flows in and out of the lungs by age 25^37^.

Although the present results must be considered preliminary in nature for reasons that include the small numbers of cases that have examined to date, as well as the fact that all of the diseased lungs all came from persons with very severe (GOLD 4) COPD. Recent study showed hypo-cellularity in airway wall tissue of mild-to-moderate COPD patients compared with non-smokers^31^, suggesting that total cellularity in airway wall tissue might be different between mild-to-moderate and severe COPD patients. We also did not collect information about medication including inhaled or oral corticosteroids that might affect pathological findings of this study. In addition, because the fixation process used to obtain the microCT studies rendered the tissue unsuitable for either the histology or gene expression profiling studies, the histology and gene expression profiling studies were examined on companion samples of lung located adjacent to those examined by microCT. In future studies, the majority of these problems will be corrected using a novel approach recently reported by Vasilescu *et al*.^38^ from our group that will allow the histology and gene expression profiling studies to be performed on exactly the same samples of tissue.

In summary, we provided histological evidence that the bronchiolar and alveolar tissues were extensively remodeled and partially destroyed before the emphysematous lesions can be detected on thoracic MDCT scans. In addition, a large percentage of the 127 gene expression signature for emphysematous destruction were also enriched in the expression signatures associated with macrophage, CD4, CD8, and B cell lymphocyte infiltration. Finally, the analysis of genes known to be expressed by NK, LTi, and ILC innate immune cells has generated the intriguing hypothesis that innate immune stimulates the adaptive immune system to respond antigens that are already present in the lungs. Because these preliminary results were came from only 4 COPD lungs and 2 to 4 control lungs, our findings would be more robust if the number of cases were greater.

## Methods

Informed consent was obtained directly from patients on the waiting list for treatment of very severe (GOLD 4) COPD by lung transplantation at the University of Pennsylvania Hospital (n = 4) and from the next of kin of organ donors (n = 4) by the Gift of Life program in Philadelphia whose lungs served as controls when they were considered unsuitable for transplantation^11, 15^. This study was approved by the institutional review boards (the human ethics approval certificate number from the Providence Health Care Research Ethics Board at St. Paul’s Hospital: H07-00211, and the University of British Columbia Biosafety certificate number: B15-0018) and conforms to the Helsinki Declaration. Movement of lung tissue and related clinical data between institutions was conducted under terms negotiated in material transfer agreements between the institutions involved that were compliant with the US Health Insurance Portability and Accountability Act (HIPAA). Each of these lungs was inflated with air, frozen solid in liquid nitrogen vapor, and kept frozen on dry ice while an MDCT scan was obtained. The frozen specimen was then cut into contiguous 2-cm thick transverse slices from lung apex to base, and 32 sites sampled in lungs from 4 patients with the centrilobular emphysematous phenotype of COPD were compared to 29 sites sampled from 4 unused donor (control) lungs.

### Sampling procedures

The cellular and molecular events associated with the initiation and progression of centrilobular emphysema was examined using a sampling system intentionally biased towards least diseased regions of lungs from patients treated for very severe (GOLD 4) COPD in order to compare regions of the diseased lungs where the mean linear intercept (Lm), used to monitor the level of emphysematous destruction in COPD, remained within the range observed in control lungs (mean Lm = 424.0 ± 48.0 µm, range 225–600 µm with an upper 95% confidence interval of 482 µm) to other regions of the diseased lungs where Lm was either between 600–1000 µm or ranged up to and beyond 1000 µm where the emphysematous lesions first become large enough to be visible on thoracic MDCT scans.

### MicroCT

One complete set of samples (n = 61) previously examined by microCT using procedures described in detail elsewhere^11, 15^ provided the number of terminal bronchioles/ml lung as well as microCT measurements of Lm measured at 20 different levels in each tissue core beginning with a random start^11^.

### Quantitative Histology

The total volume of tissue present in each core (n = 61) was measured from the electronic record of the microCT scans and the Vv of bronchiolar and alveolar tissues present in each sample as well as the Vv of the bronchial and alveolar tissue occupied by total collagen, collagen I, collagen III, elastin, infiltrating macrophages, PMNs, eosinophils, CD4 cells, CD8 cells, B cells, and NK cells was established by point counting appropriately stained histology slides (see Supplementary Table S1 for a complete list of stains and antibodies used) using a multi-level sampling design consistent with ATS/ERS guidelines^39^. In addition, tertiary lymphoid organs (lymphoid follicles) present in these lung tissues was quantified by computing the percentage of airways and vessels that contain the lymphoid collections as previously described^5^.

### Gene expression profiling

The gene expression profiling data used in this study has been reported previously^15^, and was available on 6 of the 8 cases (i.e., 2 controls and 4 subjects with centrilobular emphysema) (Gene Expression Omunibus; GSE27597, sample numbers 6982 and 6983 as control lungs and sample numbers 6965, 6968, 6969, and 6971 as COPD lungs).

### Data analysis

The remodeling of the collagen and elastin in bronchiolar and alveolar tissue, as well as the accumulated volumes of each infiltrating cell present in these tissues, was examined using both a linear mixed-effects model^15^ and a random forest analysis with the Boruta feature selection^14^. In addition, the approach used by Campbell *et al*.^15^ to determine the gene expression signature associated with emphysematous lung destruction (i.e. a progressive increase in Lm) was used to determine the gene expression profiles associated with each infiltrating inflammatory immune cell into the lung tissue. Further, because the ILC cells are very difficult to demonstrate by immunohistochemistry, GSEA^16^ was used to determine if the gene expression signatures associated with infiltrating PMNs, macrophages, CD4 cells, CD8 cells, and B cells were enriched by genes in the published gene sets associated with ILC1, ILC2, ILC3, LTi, NK innate immune cells^40–42^ and dendritic cells^43–45^. A complete list of the genes expressed by each of the cells examined can be found in Supplementary Table S2.

## Electronic supplementary material

Supplementary Information
