# Supplementary material for: The cellular and molecular determinants of emphysematous destruction in COPD
Source: Sci Rep. 2017 Aug 25;7:9562. doi: 10.1038/s41598-017-10126-2 (PMC5573394; doi:10.1038/s41598-017-10126-2)
Supplement: Supplementary file 1 — Supplementary Information [file 41598_2017_10126_MOESM1_ESM.pdf]

# The cellular and molecular determinants of emphysematous destruction in COPD

Masaru Suzuki, Marc A. Sze, Joshua D. Campbell, John F. Brothers II, Marc E. Lenburg, John E. McDonough, W. Mark Elliott, Joel D. Cooper, Avrum Spira, James C. Hogg

Online data supplement

**Methods**

Informed consent was obtained from patients waiting for treatment of very severe (GOLD 4) COPD by lung transplantation (n=4) and from the next of kin of organ donors (n=4) whose lungs served as controls when considered unsuitable for transplantation (1, 2). Each lung was inflated with air, frozen solid in liquid nitrogen vapor, and kept frozen on dry ice while a volumetric multi-detector computed tomography (MDCT) scan was obtained. The specimen was then kept frozen while cut into contiguous 2-cm thick transverse slices from lung apex to base, and 32 sites sampled in lungs from patients with the centrilobular emphysematous phenotype of COPD were compared to 29 sites sampled from unused donor (control) lungs. The sampled sites were registered in the electronic record of the specimen MDCT scan as previously described (1).

**MicroCT:** One complete set of samples (n=61) was examined by microCT as reported elsewhere (1, 2). The product of the mean number of terminal bronchioles per milliliter of each lung, as measured on microCT, and the total lung volume, as measured on MDCT of the same lung, provided an estimate of the total number of terminal bronchioles per lung or lung pair. The product of the total number of terminal bronchioles per lung and the average crosssectional area provided the total cross-sectional area of all terminal bronchioles in each lung. The mean linear intercept (Lm), which has a direct linear relationship with air-space size, was measured from images captured at 20 regularly spaced intervals within the microCT scans of each sample with the use of a previously validated grid of test

lines projected onto the image and a custom macro (Image Pro Plus, Media Cybernetics, Rockville, MD, USA). Based on the assumption that the alveoli have a spherical shape, the ratio of the surface area / volume is

$\frac{surface\ area}{Volume} = \frac{4\pi r^2}{\frac{4}{3}\pi r^3}$, which simplifies to Surface area = $\frac{4x\ sphere\ Volume}{4/3r} = \frac{4xVolume}{2/3D}$ $and\ \frac{2}{3}D = Lm.$

Therefore, the linear measurement of Lm is linked to the ratio 4 × volume /surface area of a sphere in a manner similar to the way that the numerical constant Pi (π) is defined by the ratio of the circumference of a circle to its diameter.

**Quantitative Histology**: Portions of the companion samples (n=61) to those examined by microCT, were warmed to -1˚C, and vacuum embedded in a 50% mixture of Tissue-Tek O.C.T. (Sakura Finetek USA Inc, Torrance, CA, USA) in 10% sucrose over a period of approximately 2 minutes, and immediately refrozen on dry ice. Each of these frozen samples was then mounted in a cryostat and serial histological sections cut from them were assigned to either quantitative histology or gene expression profiling. A multi-level sampling design consistent with ATS/ERS guidelines (3), in which the reference volume for each lung (n=8) was computed from the MDCT specimen scan and the reference volumes of each individual sample (n=61) from the microCT scans of these samples, with the exception that we abandoned the stratified uniform random sampling system recommended by the ATS/ERS guidelines to focus on the development of the bronchiolar and alveolar lesions that occur before the mean linear intercept (Lm) used to estimate the size of emphysematous lesions increased to the point where they could be detected on

thoracic MDCT scans. The total volume of bronchiolar and alveolar tissue present in each sample was computed as the product of their area fractions (Vvs) measured from digital images of hematoxylin and eosin (H&E) stained histology sections by a point counting procedure, and the reference volume of the sample. The sub-volumes of bronchiolar and alveolar tissues occupied by total collagen, collagen I, collagen III, elastin, macrophages, polymorphonuclear leukocytes (PMNs), eosinophils, CD4 cells, CD8 cells, B cells, and natural killer (NK) cells were determined; as the product of their area fraction on appropriately stained digital images (see online supplementary table S1 for complete list of stains and antibodies used) to identify these different structures and cells. In addition, the numbers of lymphoid follicles present in these lung tissues were obtained by computing the percentage of airways and vessels that contain these lymphoid collections using a method described elsewhere (4).

**Gene expression profiling:** Unstained frozen tissue samples adjacent to frozen lung cores examined by histology (n=61) were used to isolate the high molecular weight (i.e. the mRNA-containing fraction) of RNA using the miRNeasy Mini Kit (Qiagen, Valencia, CA, USA). RNA integrity was assessed using an Agilent 2100 Bioanalyzer and RNA purity was assessed using a NanoDrop spectrophotometer. As previously described RNA (1 μg) was hybridized onto the Human Exon 1.0 ST array (Affymetrix Inc., Santa Clara, CA, USA) according to the manufacturer's protocol (1). Expression ConsoleVersion 1.1 (Affymetrix Inc.) was used to generate transcript-level gene expression estimates for the 'core' exon probe sets via the robust multichip average (RMA) algorithm. Gene symbols of transcript

IDs were retrieved using DAVID. The gene expression profiling data used in this study was available on 6 of the 8 cases (i.e., 2 controls (#2 and #3) and 4 subjects with centrilobular emphysema) reported here. These data are available through the Gene Expression Omnibus (GEO) under the accession GSE27597. Gene expression profiling data was not available on the two additional control cases (controls #1 and #4) examined by microCT and histology in this study.

**Data analysis:** The remodeling of the bronchiolar and alveolar tissue and infiltration of these tissues by inflammatory immune cells was examined in relation to microCT measurements of emphysematous destruction using a linear mixed-effects model (1). In addition, a random forest analysis with the Boruta feature selection (5) was used to determine which of the microCT and histological measurements best predicted the increase in Lm. The Boruta program iteratively compares importances of attributes with importances of shadow attributes, created by shuffling original ones. Attributes that have significantly worst importance than shadow ones are being consecutively dropped. On the other hand, attributes that are significantly better than shadows are admitted to be confirmed. The importance measure values were obtained by the average of Z scores of the number of random forest runs. 28 variables as shown below were put into the Boruta feature selection.

- Number of terminal bronchioles
- Small airway thickness
- Vv of macrophages in the alveolar tissue (alveolar wall + alveolar space)
- Vv of macrophages in the alveolar wall

- Vv of macrophages in the alveolar space
- Vv of macrophages in the bronchiolar tissue
- Averaged Vv of macrophages in the alveolar and bronchiolar tissues
- Vv of PMNs in the alveolar tissue (alveolar wall + alveolar space)
- Vv of PMNs in the bronchiolar tissue
- Averaged Vv of PMNs in the alveolar and bronchiolar tissues
- Vv of B cells in the alveolar tissue (alveolar wall + alveolar space)
- Vv of B cells in the bronchiolar tissue
- Averaged Vv of B cells in the alveolar and bronchiolar tissues
- Vv of CD4 cells in the alveolar tissue (alveolar wall + alveolar space)
- Vv of CD4 cells in the bronchiolar tissue
- Averaged Vv of CD4 cells in the alveolar and bronchiolar tissues
- Vv of CD8 cells in the alveolar tissue (alveolar wall + alveolar space)
- Vv of CD8 cells in the bronchiolar tissue
- Vv of NK cells in the alveolar tissue (alveolar wall + alveolar space)
- Vv of NK cells in the bronchiolar tissue
- Vv of eosinophils in the alveolar tissue (alveolar wall + alveolar space)
- Vv of eosinophils in the bronchiolar tissue
- Vv of total collagen in the alveolar wall
- Vv of total collagen in the bronchiolar tissue
- Vv of collagen I in the alveolar wall
- Vv of collagen I in the bronchiolar tissue

- Vv of collagen III in the alveolar wall
- Vv of collagen III in the bronchiolar tissue

In addition, the approach reported by Campbell et al. to determine a 127 gene expression profile for emphysematous destruction (1) was applied to determine if the gene expression profiles associated with each individual infiltrating inflammatory immune cell by substituting the volume fraction (Vv) of tissue occupied by the inflammatory immune cell under study for Lm in equation 2 of the model described in reference 1.

1. $Gene_{ij} = \beta_0 + \beta_{Slice}*Slice_{ij} + \alpha_j + \varepsilon_{ij}$

2. $Gene_{ij} = \beta_0 + \beta_{Slice}*Slice_{ij} + \beta_{Vv}*Vv_{ij} + \alpha_j + \varepsilon_{ij}$

$i$=1,2,…,8; $j$=1,2,…,8

$\varepsilon_{ij} \sim N(0, \sigma^2)$, $\alpha_j \sim N(0, \sigma^2_{\alpha j})$

$Gene_{ij}$ is the $\log_2$ expression value for sample $i$ in patient $j$ for a single gene. Slice is a fixed effect controlling for the position within the lung from which the sample core was obtained. The random term $\varepsilon_{ij}$ represents the random error which was assumed to be normally distributed, $\alpha_j$ represents the random effect for patient, and $\beta_0$ represents the intercept. Both equations were then solved and if the solution for model 2 explained the data better than model 1 as determined by a significant p-value from a likelihood ratio test between the two models and the application of a false discovery rate (FDR) correction, that gene was said to be associated with that infiltrating cell. We chose a threshold of FDR<0.10 because we followed the same threshold we used in the previous study that reported 127 gene expression signature for emphysematous destruction (1). A chi-square test was then used to

determine if any of the genes in the profile significantly associated with each individual infiltrating cell was present in the 127 genes expression signature for emphysematous destruction (1). All statistical analyses were conducted using R statistical software ver. 2.9.2 on Windows and Mac OS with the "nlme" and the "Boruta" packages. In addition, Gene Set Enrichment Analysis (GSEA) (6) was used to determine if the gene expression signatures associated with the number of terminal bronchioles, Lm, and PMN, macrophage, CD4, CD8, and B cell infiltrations were enriched by genes in either the published gene expression profiles for ILC1, ILC2, ILC3, LTi or NK innate immune cells (7-9) and dendritic cells (10-12) where we had no histological information about the Vv of the infiltrating cells. A complete list of the genes expressed by all of the cells examined can be found in Supplementary Table S2.

## References


1. Campbell, J. D., *et al.* A gene expression signature of emphysema-related lung destruction and its reversal by the tripeptide GHK. *Genome Med.* **24,** 67 (2012).
2. McDonough, J. E., *et al.* Small-airway obstruction and emphysema in chronic obstructive pulmonary disease. *N. Engl. J. Med.* **365,** 1567-1575 (2011).
3. Hsia, C. C., Hyde, D. M., Ochs, M., Weibel, E. R. & ATS/ERS Joint Task Force on Quantitative Assessment of Lung Structure. An official research policy statement of the American Thoracic Society/European Respiratory Society: standards for quantitative assessment of lung structure. *Am. J. Respir. Crit. Care Med.* **181,** 394-418 (2010).
4. Hogg, J. C., *et al.* The nature of small-airway obstruction in chronic obstructive pulmonary disease. *N. Engl. J. Med.* **350,** 2645-2653 (2004).
5. Krusa, M. B. & Rudnicki, W. R. Feature selection with the Boruta package. *J. Stat. Softw.* **36,** 1-13 (2010).
6. Subramanian, A., *et al.* Gene set enrichment analysis: a knowledge-based approach for interpreting genome-wide expression profiles. *Proc. Natl. Acad. Sci. U. S. A.* **102,** 15545-15550 (2005).
7. Spits, H., *et al.* Innate lymphoid cells--a proposal for uniform nomenclature. *Nat. Rev. Immunol.* **13,** 145-149 (2013).
8. Monticelli, L. A., Sonnenberg, G. F. & Artis, D. Innate lymphoid cells: critical regulators of allergic inflammation and tissue repair in the lung. *Curr. Opin. Immunol.* **24,** 284-289 (2012).

9. Philip, N. H. & Artis, D. New friendships and old feuds: relationships between innate lymphoid cells and microbial communities. *Immunol. Cell Biol.* **91,** 225-231 (2013).
10. Dendritic cells in regulating TH1 and TH2 development. Pathway information provided by BioCarta. http://cgap.nci.nih.gov/Pathways/BioCarta/h_dcPathway (2016).
11. Tsoumakidou, M., Demedts, I. K., Brusselle, G. G. & Jeffery, P. K. Dendritic cells in chronic obstructive pulmonary disease: new players in an old game. *Am. J. Respir. Crit. Care Med.* **177,** 1180-1186 (2008).
12. Condon, T. V., Sawyer, R. T., Fenton, M. J. & Riches, D. W. Lung dendritic cells at the innate-adaptive immune interface. *J. Leukoc. Biol.* **90,** 883-895 (2011).

**Table S1. Antibodies for immunohistochemistry**

| Target | Antibody name | Antibody type | Clone | Supplier | Dilution | Fixation |
|---|---|---|---|---|---|---|
| Type I collagen | Type I collagen | Rabbit polyclonal | - | Biogenesis | 1:200 | Acetone |
| Type III collagen | Type III collagen | Rabbit polyclonal | - | Biogenesis | 1:200 | Acetone |
| Elastin | Elastin | Mouse monoclonal | BA-4 | Novocastra | 1:100 | 10% formalin |
| Macrophages | CD68 | Mouse monoclonal | EBM11 | DAKO | 1:700 | 10% formalin |
| CD4 cells | CD4 | Mouse monoclonal | MT310 | DAKO | 1:50 | 10% formalin |
| CD8 cells | CD8 | Mouse monoclonal | C8/144B | DAKO | 1:50 | 10% formalin |
| B cells | CD79a | Mouse monoclonal | JCB117 | DAKO | 1:50 | Acetone |
| NK cells | Natural killer cell-like | Mouse monoclonal | NK-1 | DAKO | 1:100 | 10% formalin |
| PMNs | Neutrophil elastase | Mouse monoclonal | NP57 | DAKO | 1:75 | 10% formalin |

* Eosinophils were identified by Hansel stain.

**Table S2. List of genes used for innate lymphoid cell subtypes in GSEA analysis**

| NK cells | LTi cells | ILC1 | ILC2 | ILC3 | DC |
|---|---|---|---|---|---|
| EOMES | AHR | ICOS | CRTH2 | AHR | ANPEP |
| GITR | ID2 | ID2 | GATA3 | ICOS | CD2 |
| ID2 | IL1B | IFNG | ICOS | ID2 | CD5 |
| IFNG | IL7 | IL12 | ID2 | IL1B | CD7 |
| IL12 | IL17 | IL18 | IL5 | IL22 | CD33 |
| IL15 | IL22 | IL1R | IL7 | IL1R | CD40 |
| IL18 | IL1R | IL7R | IL13 | IL7R | CD80 |
| IL2RA | IL7R | IL12RB2 | IL25 | IL23R | CD83 |
| IL7R | IL23R | KLRB1 | IL33 | KIT | CD86 |
| IL12RB2 | KIT | RORC | IL1R | KLRB1 | CD209 |
| KLRB1 | KLRB1 | TBX21 | IL7R | NCAM1 | CSF2 |
| LFA1 | RORC | | IL17RB | NCR1 | IFNA1 |
| NCAM1 | | | KIT | NKp44 | IFNB1 |
| NCR1 | | | KLRB1 | RORC | IL10 |
| NCR2 | | | RORA | | IL12A |
| TBX21 | | | ST2 | | IL12B |
| | | | TSLP | | IL13 |
| | | | | | ITGAX |
| | | | | | LAMP3 |
| | | | | | TLR2 |
| | | | | | TLR4 |
| | | | | | TLR7 |
| | | | | | TLR9 |

LTi = lymphoid tissue-inducer, ILC = innate lymphoid cell, DC = dendritic cells.

## Table S3. Quantitative histology of the severity of emphysema to inflammatory cell infiltration and tissue remodeling

| | Bronchiolar tissue | | | | Alveolar tissue | | | |
|---|---|---|---|---|---|---|---|---|
| | Control | COPD | | | Control | COPD | | |
| | Lm≤482 µm | Lm≤600 µm | 600<Lm≤1000 µm | Lm>1000 µm | Lm≤482 µm | Lm≤600 µm | 600<Lm≤1000 µm | Lm>1000 µm |
| **Alveolar surface area ($cm^2$)** | - | - | - | - | 471.3 ± 16.3 | 323.9 ± 23.5* | 199.4 ± 7.9*† | 108.6 ± 11.5*† |
| **Small airway wall thickness (mm)** | 0.06 ± 0.00 | 0.15 ± 0.01* | 0.13 ± 0.01* | 0.12 ± 0.01* | - | - | - | - |
| **# Terminal bronchioles/ml lung** | 6.95 ± 0.73 | 0.15 ± 0.15* | 0.51 ± 0.22* | 1.02 ± 0.55* | - | - | - | - |
| **Vv of macrophages ($x10^{-2}$)** | 3.09 ± 0.26 | 4.63 ± 1.01 | 6.77 ± 1.00* | 5.54 ± 1.18 | 5.78 ± 0.33 | 5.18 ± 0.89 | 9.97 ± 1.30*† | 17.6 ± 1.69*† |
| **Vv of CD4 cells ($x10^{-2}$)** | 1.55 ± 0.16 | 1.11 ± 0.65 | 3.13 ± 0.76* | 3.82 ± 0.69*† | 0.62 ± 0.05 | 0.70 ± 0.39 | 2.84 ± 0.49*† | 2.48 ± 0.37*† |
| **Vv of CD8 cells ($x10^{-2}$)** | 0.22 ± 0.07 | 0.60 ± 0.17 | 0.97 ± 0.42 | 0.62 ± 0.20 | 0.11 ± 0.04 | 0.82 ± 0.31 | 1.31 ± 0.35* | 0.48 ± 0.16 |
| **Vv of B cells ($x10^{-2}$)** | 0.02 ± 0.01 | 0.52 ± 0.13 | 0.47 ± 0.17 | 0.75 ± 0.26* | 0.00 ± 0.00 | 0.13 ± 0.08 | 0.64 ± 0.16*† | 0.69 ± 0.14*† |
| **Vv of eosinophils ($x10^{-2}$)** | 0.02 ± 0.01 | 0.00 ± 0.00 | 0.18 ± 0.09 | 0.14 ± 0.08 | 0.02 ± 0.01 | 0.20 ± 0.08* | 0.29 ± 0.04* | 0.21 ± 0.04* |
| **Vv of NK cells ($x10^{-2}$)** | 0.30 ± 0.04 | 0.30 ± 0.04 | 0.64 ± 0.25 | 0.32 ± 0.15 | 0.17 ± 0.03 | 0.20 ± 0.10 | 0.59 ± 0.16 | 0.21 ± 0.08 |
| **Vv of PMNs ($x10^{-2}$)** | 3.22 ± 0.63 | 1.35 ± 0.55 | 2.42 ± 0.56 | 1.87 ± 0.44 | 7.98 ± 0.97 | 4.12 ± 1.06 | 3.45 ± 0.71 | 4.00 ± 0.66 |
| **Vv of total collagen** | 63.6 ± 2.97 | 21.3 ± 1.53* | 32.7 ± 4.93* | 37.8 ± 7.12* | 38.5 ± 2.73 | 17.9 ± 3.13 | 23.3 ± 3.28 | 28.3 ± 4.69 |
| **Vv of collagen I** | 6.60 ± 1.20 | 4.10 ± 1.67 | 17.4 ± 4.29 | 20.7 ± 8.29 | 2.62 ± 0.49 | 5.67 ± 1.68 | 12.5 ± 2.15 | 12.6 ± 3.39*† |
| **Vv of collagen III** | 38.1 ± 3.40 | 6.42 ± 3.89 | 13.4 ± 4.95 | 12.7 ± 5.97* | 25.8 ± 2.38 | 3.84 ± 2.34 | 10.3 ± 3.39 | 10.3 ± 2.76 |
| **log (collagen I/III ratio)§** | -1.85 ± 0.29 | 0.55 ± 1.65 | 2.01 ± 0.79† | 0.63 ± 0.60* | -2.28 ± 0.26 | 0.97 ± 0.63* | 1.02 ± 0.44* | 0.12 ± 0.36* |
| **Vv of elastin** | 7.87 ± 1.24 | 2.51 ± 1.30 | 2.40 ± 0.63* | 4.44 ± 1.12 | 6.00 ± 0.54 | 14.2 ± 3.34* | 11.8 ± 1.48* | 18.5 ± 3.05*‡ |

Vv = volume fraction. Mean±SEM. * $p<0.05$ versus control, †$p<0.05$ versus Lm≤600 µm, ‡ $p<0.05$ versus 600<Lm≤1000 µm.

§ Collagen I/III ratio was log-transformed to normalize the distribution. Minus value means a relative increase in collagen III compared to collagen I

**Table S4. Multivariate linear mixed-effects model for the association with the increase in Lm using significant variables by the Boruta feature selection**

| Variable tested | Coefficient β (95% CI) | Significance |
|---|---|---|
| Vv macrophages (alveolar wall) | 3.61 (0.78-6.45) | 0.01 |
| Vv CD4 cells (alveolar tissue) | 6.53 (0.0004-13.1) | 0.05 |
| Vv B cells (alveolar tissue) | 33.4 (10.4-56.4) | 0.006 |
| Vv B cells (bronchiolar tissue) | -0.96 (-21.5-19.6) | 0.92 |
| Vv eosinophils (alveolar tissue) | -32.6 (-101.0-35.9) | 0.34 |
| Vv collagen I (alveolar wall) | 0.49 (-1.08-2.07) | 0.53 |
| Number of terminal bronchioles /ml | -0.0001 (-0.03-0.03) | 0.99 |

CI = confidence interval, Coefficient β = the mathematical weightings of the explanatory variables. In order to avoid multicolinearity, variables from averaged or summed values were not put into the multivariate models.

**Table S5. Genes associated with alveolar infiltration of macrophages, CD4 cells, and B cells overlapped with 127 gene expression signature for emphysematous destruction**

| Gene | Macrophages | CD4 cells | B cells |
|---|---|---|---|
| ACVRL1 | Negative | - | Negative |
| ADRB1 | Negative | - | - |
| ARHGEF10 | Negative | - | - |
| ATOH8 | - | Negative | - |
| BCL11A | Positive | - | Positive |
| C13orf15 | Negative | - | - |
| C8orf34 | - | - | Positive |
| CCR7 | Positive | - | Positive |
| CD22 | - | Positive | - |
| CD79A | - | Positive | Positive |
| CTTNBP2NL | Negative | - | - |
| CXCL13 | - | - | Positive |
| DHRS9 | - | Positive | Positive |
| EDNRB | Negative | - | - |
| ENG | Negative | - | - |
| EPAS1 | Negative | - | - |
| FAIM3 | - | Positive | Positive |
| FCN3 | Negative | - | - |
| FCRLA | - | Positive | - |
| FOXF1 | Negative | - | Negative |
| GPR110 | - | - | Positive |
| IRF4 | Positive | Positive | - |
| KIAA0125 | Positive | Positive | Positive |
| KL | Negative | - | - |
| KLF13 | Negative | - | - |
| KLHL6 | - | Positive | Positive |
| LOC130576 | Positive | - | - |
| LPHN2 | Negative | - | - |
| MAN1C1 | - | Positive | - |

| | | | |
|---|---|---|---|
| MAOA | Negative | - | - |
| MAP2 | Negative | - | - |
| MGC29506 | Positive | Positive | - |
| MYH9 | - | Negative | - |
| OSBPL3 | - | Positive | Positive |
| PAPSS2 | Negative | - | - |
| PECAM1 | Negative | - | - |
| PHLDB1 | Negative | - | - |
| PNMAL1 | - | Positive | - |
| PRKCE | Negative | - | - |
| QKI | Negative | - | - |
| RALGPS2 | Positive | - | - |
| RBP5 | Positive | - | - |
| S100A8 | - | - | Negative |
| S1PR1 | Negative | - | - |
| SH3BP5 | Negative | - | - |
| SMAD6 | - | - | Negative |
| STARD13 | Negative | - | Negative |
| STOM | Negative | - | - |
| STXBP6 | Negative | - | - |
| SYCP2L | Negative | - | - |
| SYN2 | - | - | Negative |
| TGFBR2 | Negative | - | - |
| TMEM2 | Negative | - | - |
| TPST2 | - | - | Negative |
| UGT8 | - | - | Positive |
| VIPR1 | Negative | - | - |
| WFDC1 | Negative | Negative | Negative |
| WNT2B | Negative | - | - |

“Positive” means the positive correlation between Vv of each cell type and each gene expression. “Negative” means the negative correlation between Vv of each cell type and each gene expression.

## Supplementary figure legends

**Figure S1.** Immunostained alveolar (top panel) and bronchiolar (bottom panel) elastin (A), collagen I (B), and collagen III (C) from control (left) and very severe COPD (right). Scale bar = 200 μm.

**Figure S2.** Immunostained alveolar (top panel) and bronchiolar (bottom panel) macrophages (A), CD4 cells (B), CD8 cells (C), and B cells (D) from control (left) and very severe COPD (right). Scale bar = 200 μm.

## Figure S1

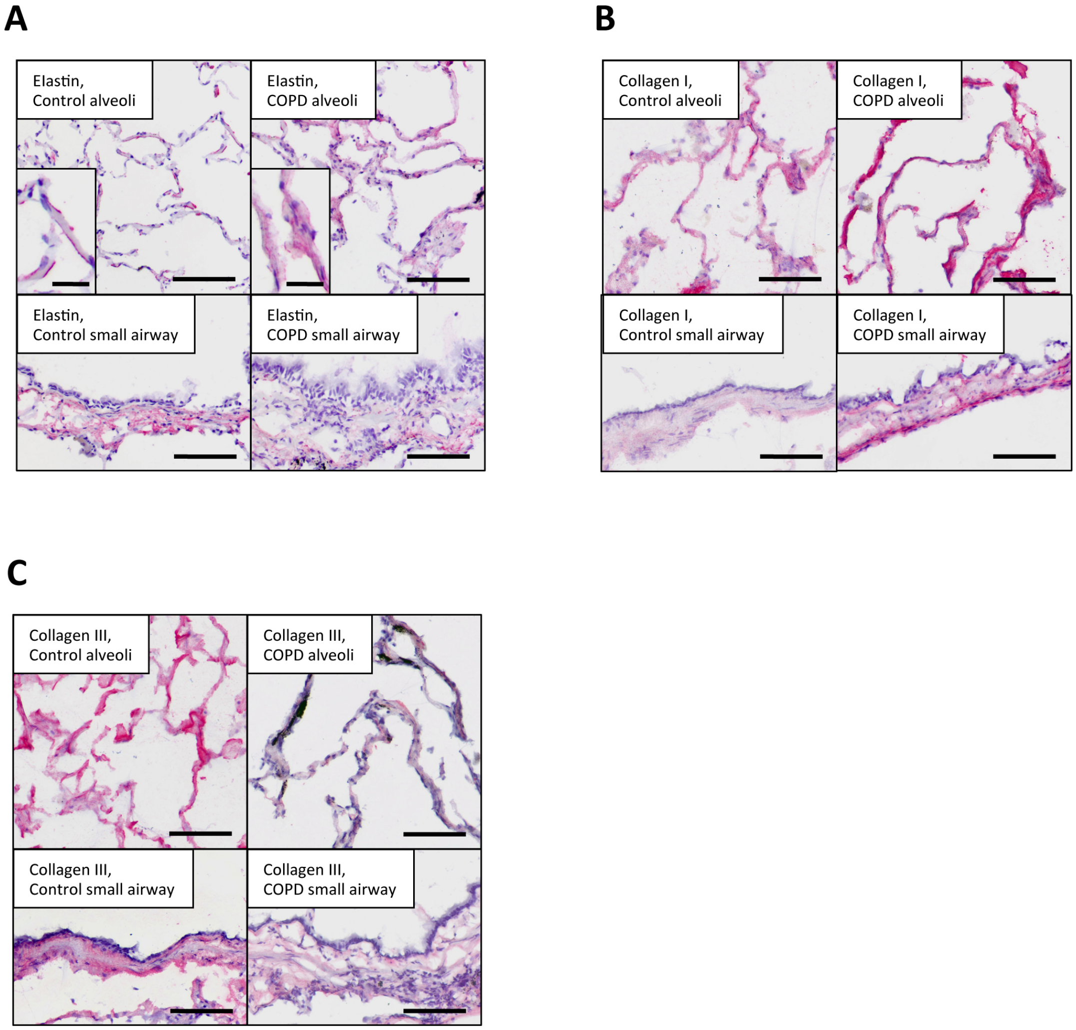
A
Elastin,
Control alveoli
Elastin,
COPD alveoli
Elastin,
Control small airway
Elastin,
COPD small airway
B
Collagen I,
Control alveoli
Collagen I,
COPD alveoli
Collagen I,
Control small airway
Collagen I,
COPD small airway
C
Collagen III,
Control alveoli
Collagen III,
COPD alveoli
Collagen III,
Control small airway
Collagen III,
COPD small airway

# Figure S2

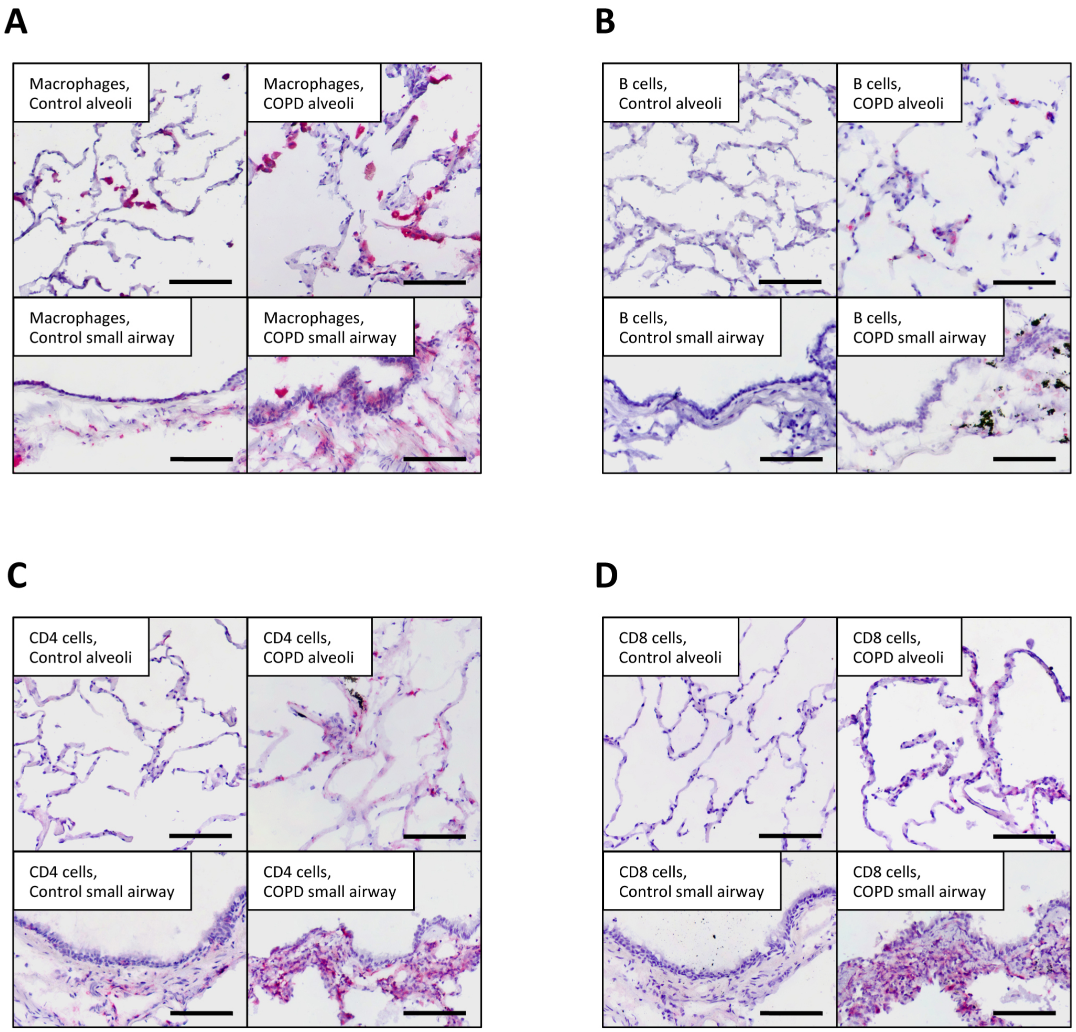
A
Macrophages, Control alveoli
Macrophages, COPD alveoli
Macrophages, Control small airway
Macrophages, COPD small airway
B
B cells, Control alveoli
B cells, COPD alveoli
B cells, Control small airway
B cells, COPD small airway
C
CD4 cells, Control alveoli
CD4 cells, COPD alveoli
CD4 cells, Control small airway
CD4 cells, COPD small airway
D
CD8 cells, Control alveoli
CD8 cells, COPD alveoli
CD8 cells, Control small airway
CD8 cells, COPD small airway
